# Preparation of a Polypyrrole-Polyvinylsulphonate Composite Film Biosensor for Determination of Cholesterol Based on Entrapment of Cholesterol Oxidase

**DOI:** 10.3390/s90806435

**Published:** 2009-08-20

**Authors:** Fadime Yıldırımoğlu, Fatma Arslan, Servet Çete, Ahmet Yaşar

**Affiliations:** 1 Department of Chemistry, Institute of Sciences, Gazi University, Ankara, Turkey; 2 Department of Chemistry, Faculty of Arts and Sciences, Gazi University, Ankara, Turkey

**Keywords:** cholesterol biosensor, amperometry, polypyrrole (PPy), polyvinylsulphonate (PVS), entrapment, interference effect

## Abstract

In this paper, a novel amperometric cholesterol biosensor with immobilization of cholesterol oxidase on electrochemically polymerized polypyrrole–polyvinylsulphonate (PPy–PVS) films has been accomplished via the entrapment technique on the surface of a platinum electrode. Electropolymerization of pyrrole and polyvinylsulphonate on the Pt surface was carried out by cyclic voltammetry between −1.0 and +2.0 V (vs. Ag/AgCl) at a scan rate of 100 mV upon the Pt electrode with an electrochemical cell containing pyrrole and polyvinylsulphonate. The amperometric determination is based on the electrochemical detection of H_2_O_2_ generated in the enzymatic reaction of cholesterol. Determination of cholesterol was carried out by the oxidation of enzymatically produced H_2_O_2_ at 0.4 V vs. Ag/AgCl. The effects of pH and temperature were investigated and optimum parameters were found to be 7.25 and 35 °C, respectively. The storage stability and operational stability of the enzyme electrode were also studied. The results show that 32% of the response current was retained after 19 activity assays. The prepared cholesterol biosensor retained 43% of initial activity after 45 days when stored in 0.1 M phosphate buffer solution at 4 °C.

## 1. Introduction

In recent years various types of biosensors have been developed, some of which are already in practical use. Such sensors have been used for different applications, including health care, food and environmental monitoring [1].

Oxido-reductase enzyme electrodes constitute a large group of biosensors, accounting for over 90% of the existing amperometric enzyme-based biosensors. The side product of the flavin-oxidase enzymes reactions is usually hydrogen peroxide, formed by the enzyme-catalyzed oxidation of the analyte by dissolved molecular oxygen [2,3].

Enzyme immobilization onto the electrode surface is a crucial step in assembling amperometric biosensors [4–6]. In recent years, mediators and conducting polymers have been used as a matrix for immobilizing the enzymes [7]. Intrinsically, conducting polymers with conjugated double bonds have been regarded as attractive advanced materials for electronic devices, electrochromic displays, chemical and biochemical sensors, drug release systems, rechargeable batteries and for modifying electrode surfaces for electrical wiring of biomolecules. Different conducting polymers have been used for the immobilization of biomolecules, such as polyacetylene, polythiophene, polypyrrole (PPY), polyindole and polyaniline [7–11]. The PPY–PVS composite membrane has been proven to play a unique role as a “charge controllable membrane” in which the fixed charges are checked electrochemically by an internal electronic state [12,13].

The determination of cholesterol levels is of importance in clinical diagnosis [14] of diseases such as coronary heart disease, myocardial infarction and arteriosclerosis [15,16]. Cholesterol is a sterol found in eggs, meats, yellow cheese, and derivatives [17,18]. Biosensors for cholesterol have been used in biochemical analysis owing to their good selectivity, fast response, low cost, small size and long term stability [17,18]. Most of the literature on cholesterol biosensors has focused on diagnosing disorders [18–21].

The enzymatic reactions in the use of cholesterol oxidase (COx) as a receptor are as follows:

$$\begin{array}{c} \text{Cholesterol}+O_{2}\to \text{Cholest-}4\text{-en-}3\text{-one}+H_{2}O_{2}\;[24,26]\\ H_{2}O_{2}\to O_{2}+{2H}^{+}+{2e}^{-}\;[22,23] \end{array}$$

The cholesterol is oxidized by COx in the presence of oxygen and hydrogen peroxide is produced at the same time. The electrooxidation current of hydrogen peroxide is detected after applying an appropriate potential to the system. The major problem for amperometric detection is the overestimation of the response current due to interferences. Recently, many researchers have mentioned the inclusion of metal nanoparticles with a catalytic effect in polymer modified electrodes to develop biosensor sensibility and to reduce the overpotential applied to the amperometric biosensors [23–28].

Pt is a well-known catalyst that has a high catalytic activity for hydrogen peroxide electrooxidation [29–31]. The amperometric detection of hydrogen peroxide is normally performed anodically (e.g., oxidation at +700 mV with a Pt working electrode), but is drastically influenced by many simply oxidizable interferents typically present in real samples [32–34].

In this paper we report the immobilization of cholesterol oxidase onto PPy–PVS film via an entrapment procedure for determination of free cholesterol. Effects of the immobilization process on kinetic parameters, storage and reuse capability of the enzyme were investigated. The optimum working conditions with respect to the substrate concentrations, the pH and temperature were investigated.

## 2. Materıal and Methods

### 2.1. Instrumentation and Reagents

All electrochemical experiments carried out using an Epsilon EC electrochemical analyzer A conventional three-electrode system was equipped with a Pt plate (0.5 cm^2^) as the working electrode, an Ag/AgCl electrode (3 M KCl) as the reference electrode, and a platinum wire (diameter and length, 1 mm, 4 cm respectively) for the counter electrode. The pH values of the buffer solutions are measured with an ORION Model 720A pH-ionmeter. Temperature control was accomplished with a Grant W14 thermostat. Cholesterol oxidase (EC 1.7.3.3 from *Aspergillus niger* purified from the microorganism and with an activity of 10 unit mL^−1^) and cholesterol were purchased from Sigma. Pyrrole and PVS was supplied by Fluka. All other chemicals were obtained from Sigma. All the solutions were prepared using distilled water.

### 2.2. Preparation of Cholesterol Solution

Cholesterol is soluble in alcohol and also in water in the presence of surfactants. Solutions were prepared daily by dissolving cholesterol in isopropanol, Triton X-100, and the phosphate buffer (pH 7.0). The isopropanol, Triton X-100, phosphate buffer ratio is 10:4:86 by weight [35].

### 2.3. Preparation of Pt/PPy–PVS Film Electrode

The surface of the Pt plate electrode cleaned according to [36] was covered by the electropolymerization of pyrrole and polyvinylsulphonate [20]. The electrode was immersed in a 10 mL solution of 0.1 M pyrrole and 0.1 M polyvinylsulphonate. The solution was purged with nitrogen in order to remove the oxygen. The electropolymerization of pyrrole upon the electrode surface was performed by the cyclic voltammetric scans between −1.0 and +2.0 V at a scan rate of 100 mV/s. The electrode was washed with buffer solution after the coating process.

### 2.4. Immobilizaton of Cholesterol oxidase on Pt/PPy-PVS Film Electrode

Pt/PPy-PVS-Cholesterol oxidase film electrode was used against the Pt electrode, which was used as the counter electrode. The reference electrode was composed of Ag/AgCl. Immobilization of cholesterol oxidase was carried out by the physical entrapment approach. The concentrations of pyrrole and polyvinylsulphonate were 0.1 M respectively. 1 mL of COx enzyme (10 U/mL) is added into solution. The solution was then purged by nitrogen for the removal of oxygen before electropolymerization. Electropolymerization was performed in the mode of cyclic voltammetry to immobilize cholesterol oxidase onto the electrode. The scanned voltage range was from −1.0 to +2.0 V with a scanning rate of 100 mV/s. After the fabrication of cholesterol oxidase entrapped polypyrrole film onto the PPy–PVS working electrode was finished, the electrode was rinsed with deionized water to remove the unreacted pyrrole monomer and free cholesterol oxidase. Immobilized enzyme electrode was kept in a refrigerator at the 4 °C in phosphate buffer when it was not in use [15].

### 2.5. Amperometric Biosensor Measurements

Amperometric response studies carried out in phosphate buffer. Operational stability, storage stability, pH and temperature were determined via application of +0.4 V with respect to Ag/AgCl electrode to detect oxidation current of H_2_O_2_. After the background current reached a stable value, cholesterol solution was added to the cell using a micropipette and stirred for 10 min then the resulting current difference was recorded. Researches on operational and storage stability as well as effects of pH and temperature were carried out using 4 × 10^−5^ M concentration of cholesterol

## 3. Result and Discussion

In this study, we prepared a new cholesterole biosensor with the entrapment of cholesterol oxidase (COx) on poly(pyrrole)-polyvinylsulphonate (PPy-PVS) composite films. The parameters effecting to the performance of the biosensor were examined.

### 3.1. The Working Potential

**Figure 1.**
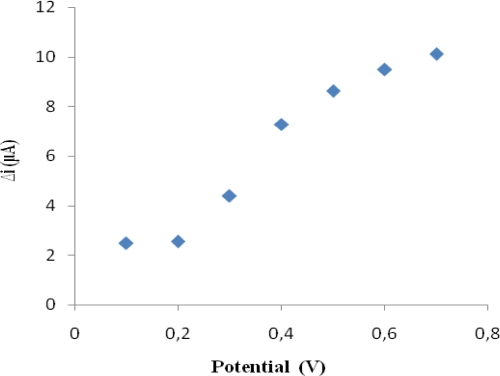
The effect of working potential on the response of the biosensor (in the phosphate buffer (pH 7.0) containing 4 × 10^−5^M cholesterol).

Current differences of H_2_O_2_ (0.1 mM) in different potentials (0.1, 0.2, 0.3, 0.4, 0.5, 0.6, 0.7 V) were measured by using Pt/PPy–PVS electrode and plotted against potential in Figure 1.

It is shown that the current of H_2_O_2_ increases until a potential of 0.4 V. At higher potential, interferences caused by exogenous substances present in body fluids (e.g., ascorbic acid) [37]. Therefore 0.4 V was used as working potential.

### 3.2. Effects of pH and Temperature

**Figure 2.**
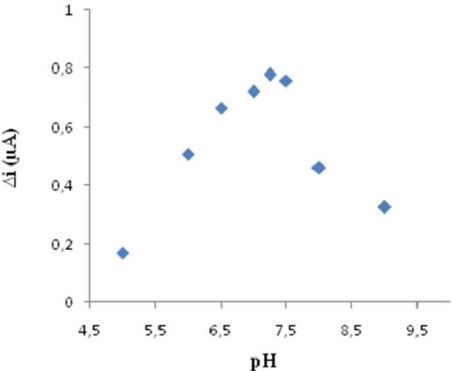
The effect of pH on the response of the biosensor (in the phosphate buffer (pH 7.0) containing 4 × 10^−5^ M cholesterol operating potential is +0.4 V).

The enzyme activity drastically depends on temperature and pH since too high or low values may inactivate the enzyme. The pH value depends on the charge of the enzyme and/or of the matrix. The pH change is useful in understanding the association between the structure and functional group of the enzyme. Extreme pH values cause enzyme denaturation. Therefore, the optimum pH values should be defined. The effect of the pH and temperature on response current of the biosensor was determined. Cholesterol oxidase is optimally efficient at 37 °C and pH 7.0 [28]. To determine the optimum pH, an assay was applied by changing the pH between 5 and 9.0 at a constant temperature. The biosensor response was increased as the pH was increased up to pH 7.25 after which it starts decreasing (Figure 2). Immobilized cholesterol oxidase enzyme showed maximum activity at pH 7.25. This value was possible. At extreme pH values the enzyme was denaturated.

**Figure 3.**
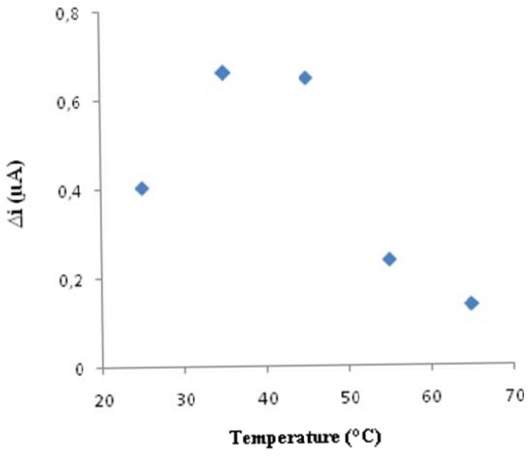
The effect of temperature on the response of the biosensor (in the phosphate buffer (pH 7.25) containing 4 × 10^−5^ M cholesterol operating potential is +0.4 V).

Temperature is an important factor which has a significant effect on enzyme activity. The biosensor response was evaluated at different incubation temperature from 25 to 65 °C. The biosensor response increased up to 25 °C (Figure 3). As illustrated in Figure 3, the current response gradually increased with increasing temperature and reached a maximum at 35 °C. Enzyme can be denaturated after a long incubation period at a temperature of 35 °C. Therefore, the temperature of 25 °C was chosen as working temperature to all further experiments.

### 3.3. Effect of Substrate Concentration on Biosensor

**Figure 4.**
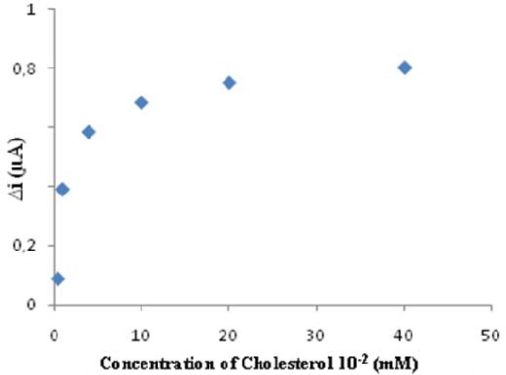
The effect of cholesterol concentration upon the amperometric response of the biosenso (Michealis-Menten plot, in the phosphate buffer (pH 7.25), operating potential is +0.4 V, 25 °C).

The value of the Michaelis–Menten kinetic parameter (K_m_), which shows the enzyme–substrate kinetics, was determined by the analysis of the slope of enzymatic reaction. V_max_ is the maximum rate for enzymatic reaction. The effect of the substrate concentration on the reaction rate, catalyzed by immobilized ChOx, was studied using varying initial concentration (5 × 10^−6^−4 × 10^−4^ M) of cholesterol substrate (Figure 4). I_max_ and K_m_ (app) were calculated from Lineweaver–Burk plots [34]. With the increase in substrate concentration, there was an increase in amperometric current signal. Kinetic parameters K_m_ (app) and I_max_ for the enzyme biosensor were detected at constant temperature (25 °C) and pH (pH 7.25) while varying the substrate concentration. K_m_ (app) and I_max_ were calculated as 40 mM, 1.17 μM/min respectively. K_m_ values for immobilized ChOx presented in the literature are 9.8, 0.41, 2.72 mM [38–40].

The K_m_ value of the system determines the affinity of enzyme for its substrate, with a smaller value of K_m_ indicating increased affinity of enzyme for its substrate. For the fabrication of biosensors, different matrices and methods of immobilization of enzymes were employed, and these could result in different conformational changes in the enzyme structures given that the enzyme kinetics is environment sensitive. Hence, the variation in value of K_m_ could be attributed to these facts [41].

### 3.4. Operational Stability and Storage Stability

**Figure 5.**
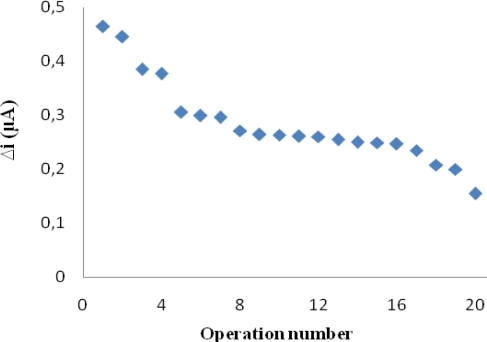
Measure number of the biosensor (in the phosphate buffer (pH 7.25), operating potential is +0.4 V, 25 °C)

**Figure 6.**
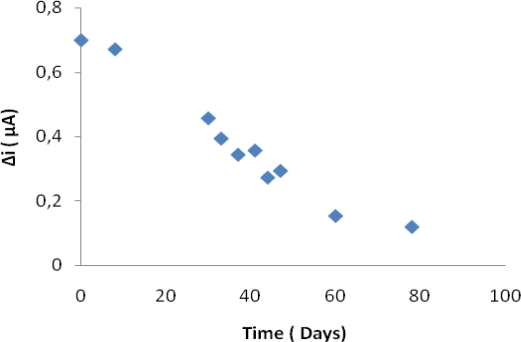
Storage stabilization of the biosensor (in the phosphate buffer (pH 7.25), operating potential is +0.4 V, 25 °C).

The biosensor was used at optimum activity conditions for 19 activity assays in one day to determine the operational stability. Storage stability of the biosensor was determined by performing activity assays within 45 days. The operational stability was studied by applying activity assay (under optimum conditions) for 19 times in the same day at constant temperature, pH and substrate concentration. At the end of the 19 measurements, the biosensor lost 68 % of its initial activity (Figure 5). The activity assay was applied within 45 days to display the storage stability of immobilized enzyme. As shown in an activity loss of 57% was observed on the 45th day (Figure 6). In general, an enzyme is not stable in aqueous solution during storage and the activity is gradually reduced [30]. The reusability was tested because of its importance for repeated applications in a batch reactor. The main advantage of reusability is to reduce the cost of the treatment.

### 3.5. Interference Effect

A few common substances found in serum or urine were studied for any interfering effect on the analysis of cholesterol. Known concentrations of ascorbic acid, glucose and paracetamol (acetoaminophen) were added and the results are shown in Table 1. It has been observed that uric acid has been no interfering effects on the analysis of cholesterol. But interfering effects of ascorbic acid, paracetamol and glucose on the analysis of cholesterol were observed. These interferences were almost removed by dilution of solution in cell.

## 4. Conclusions

In this work, cholesterol oxidase was successfully immobilized on a poly(pyrrole)-polyvinylsulphonate (PPy–PVS) composite film. The experimental results showed clearly that the biosensor exhibited good performance in the determination of cholesterol. The cholesterol biosensor has high sensitivity and good selectivity. Operational stability and long term storage stability are good. In addition, PPy–PVS can provide a biocompatible and electrochemical microenvironment for immobilization of enzyme, making this material a good candidate for the fabrication of highly sensitive and selective cholesterol biosensors.

## Figures and Tables

**Table 1.** Interfering substances on the amperometric response of the biosensor.

**Interfering substances**	**Concentration**	**Response current of interfering substances (μA)**
Glucose	5 × 10^−3^(blood)	0.063
Uric acid	1 × 10^−4^(blood)	-
Paracetamol (acetaminophen)	1 × 10^−4^(blood)	0.602
Ascorbic acid	1 × 10^−4^(blood)	0.176
